# Causal association between psycho-psychological factors, such as stress, anxiety, depression, and irritable bowel syndrome: Mendelian randomization

**DOI:** 10.1097/MD.0000000000034802

**Published:** 2023-08-25

**Authors:** Zhihao Diao, Wenchang Xu, Danyang Guo, Jingzhi Zhang, Ruiyu Zhang, Fengzhao Liu, Yufei Hu, Yuxia Ma

**Affiliations:** a School of Acupuncture and Tuina, Shandong University of Traditional Chinese Medicine, Jinan, China; b The First Clinical Medical College, Shandong University of Traditional Chinese Medicine, Jinan, China; c Affiliated Hospital of Shandong University of Traditional Chinese Medicine, Jinan, China.

**Keywords:** stress, anxiety, depression, irritable bowel syndrome, Mendelian randomization, mental and psychological factors

## Abstract

**Background:**

Pathogenesis, diagnosis, and treatment of irritable bowel syndrome (IBS) have been reported to be challenging hotspots in clinical practice. Previous observational studies have found that stress, anxiety, depression, and other mental and psychological diseases are closely associated with IBS. This study aimed to further explore the causal relationships of these associations through Mendelian randomization (MR).

**Methods:**

The data needed for MR were obtained from publicly published genome-wide association databases. We performed a bidirectional, 2-sample MR analysis using instrumental variables (IV) associated with stress, anxiety, and depression, and other mental and psychological factors as exposures and IBS as the outcome. A reverse MR analysis with IBS as exposure and stress, anxiety, depression, and other mental and psychological factors as the outcomes was also performed. The inverse variance weighting (IVW) method was adopted as the main method of MR, and the causal effect between stress, anxiety, depression, and other mental and psychological factors and IBS was evaluated as the main result of the study. In addition, a series of sensitivity analyses was conducted to comprehensively evaluate the causal relationship between them.

**Results:**

Stress, anxiety, depression, and other mental and psychological factors were the underlying etiologies for IBS (odds ratio [OR] = 1.06, 95% confidence interval [CI]: 1.03–1.08), and they were positively correlated. Univariate analysis further supported the above conclusions (Depression, [OR = 1.31, 95% CI: 1.05–1.63, *P* = .016], Anxiety, [OR = 1.53, 95% CI: 1.16–2.03, *P* = .003]). However, in reverse MR analysis, we found that IBS did not affect stress, anxiety, depression, or other mental and psychological factors and that there was no causal relationship between IBS and stress, anxiety, depression, or other mental and psychological factors (*P* > .05).

**Conclusion:**

This study demonstrates that mental and psychological factors are the underlying etiologies for IBS. These findings may provide important information for physicians regarding the clinical treatment of IBS.

## 1. Introduction

Irritable bowel syndrome (IBS) is a common functional gastrointestinal disease without organic lesions. It is chronic, recurrent, and difficult to cure, which seriously reduces the quality of people daily lives^[1]^ and causes a significant economic burden to society.^[2]^ According to research, the incidence of IBS is on the rise.^[3]^ The overall incidence of IBS is approximately 11.2% worldwide.^[4]^ The etiology of IBS is complex. It is widely believed that diet, inflammation, intestinal flora, genetic immunity, and environment are risk factors for IBS.^[5,6]^ However, there are other factors with potential pathogenicity for the onset of IBS, such as mental and psychological stress.

IBS is comorbid with stress, anxiety, depression, and other mental and psychological disorders.^[7,8]^ In addition, a meta-analysis showed that the onset, development, and treatment of IBS are closely related to stress, anxiety, and depression,^[9,10]^ among which anxiety and depression are the most common.^[11]^ A prospective study^[12]^ showed that the stressor scores of patients with IBS increased markedly before the onset of the disease. Another study found that among the observers included in the trial, the number of patients with IBS and anxiety accounted for 44%, and the incidence of IBS and depression was as high as 84%.^[13]^ At present, the treatment of IBS is primarily based on drugs and concurrent methods, including diet adjustments, psychotherapy, and appropriate exercise.^[14]^ In basic clinical research, tricyclic antidepressants and psychological intervention can effectively improve the symptoms of IBS.^[15]^ Although most observational studies have found a causal relationship between mental and psychological factors, such as stress, anxiety, and depression, and IBS, they cannot determine a causal relationship. Additionally, observational studies are prone to bias and reverse causation.^[16]^

Mendelian randomization (MR) is an emerging approach exploring causality based on genetic epidemiology. The main purpose of MR is to use genetic variants as instrumental variables (IV) in non-experimental data to estimate the association between risk factors (often replaced by the term exposure) and health or disease outcomes.^[17,18]^ The genetic variation of each person is randomly assigned by the parents, independent of confounding factors, and has been determined at the time of conception and will not change;^[19]^ therefore, bias and reverse causality caused by confounding factors can be avoided,^[20]^ and MR can compensate for the shortcomings of randomized controlled trials, such as high cost and prolonged duration. This study aimed to explore the causal relationship between stress, anxiety, depression, and other mental and psychological factors and IBS and to provide new ideas for preventing and treating IBS.

## 2. Methods

### 2.1. Study design

**Figure 1.**
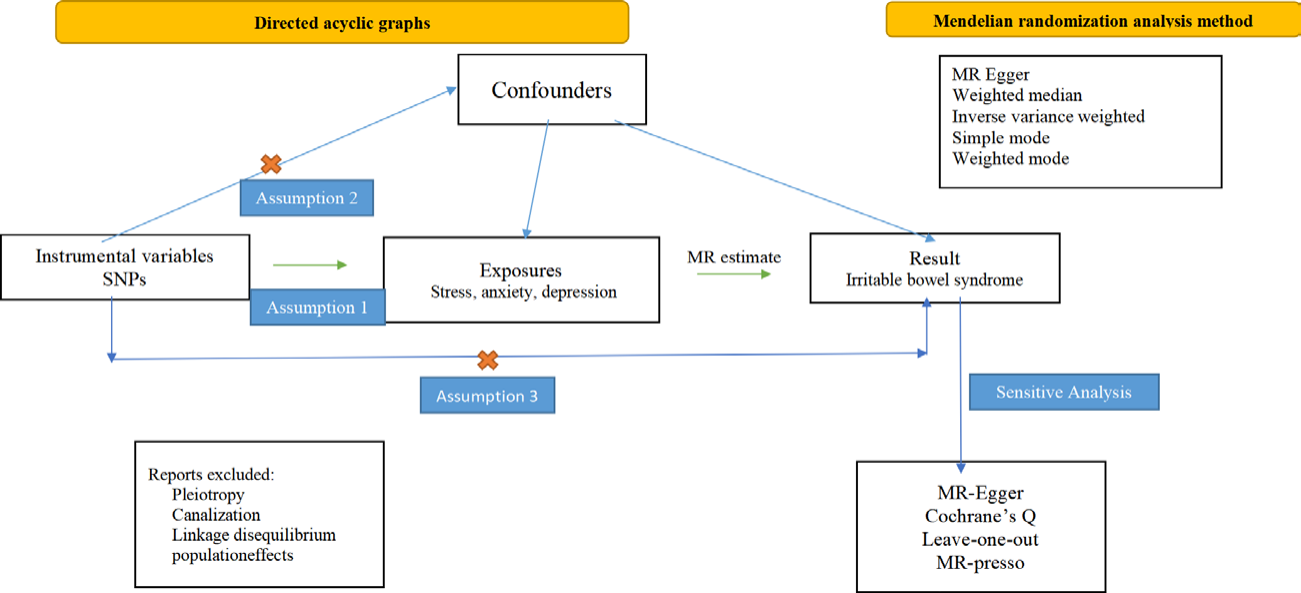
Directed acyclic graph for 2 sample Mendelian randomization studies.

To evaluate the causal relationship between mental and psychological factors, such as stress, anxiety, depression, and IBS, we summarized the genetic variants related to stress, anxiety, and depression from the genome-wide association study database as exposures and IBS as the outcome. We used the 2-sample MR Method for analysis, in which the single nucleotide polymorphism (SNP) as IV needed to meet 3 basic conditions^[21]^: the genetic variants to be associated with stress, anxiety, or depression; genetic variation to be independent of any confounding factors; and the genetic variants should not affect IBS directly but only through stress, anxiety, or depression (See Fig. 1 for details of the directed acyclic graph). In addition, we performed an inverse MR analysis with IBS as the exposure and stress, anxiety, depression, and other mental and psychological factors as outcomes.

### 2.2. Data resources

Summary data for this study were obtained from the IEU OpenGWAS project (https://gwas.mrcieu.ac.uk) and FinnGen consortium (http://finngen.fi/en). To effectively avoid the data overlap and ensure the scientific validity and reliability of the results, abstract data of stress, anxiety, depression, and other mental and psychological factors were extracted from the above 2 databases respectively. We obtained 158,565 cases and 300,995 controls in the UK Biobank, with a prevalence of 52.69%. We also extracted summary data on stress, anxiety, depression, and other mental and psychological factors (https://r8.finngen.fi/) as collected by the FinnGen Consortium from 73,610 patients and 664,862 controls. Summary data on IBS were obtained from a meta-analysis of European populations conducted by the Medical Research Council Integrated Epidemiology Unit at the University of Bristol, which included 10,939 patients with IBS and 451,994 controls. The mean age first upon diagnosis of IBS was 49.43 years. Analyses were adjusted for age, sex, major components, and genotype-batch effects. Diseases were discharge- or death-coded according to the International Statistical Classification of Diseases and Related Health Problems. The SNP data are presented in supplementary Table 1 and supplementary Table 2, http://links.lww.com/MD/J512.

### 2.3. Selection of IV

SNPs of genome-wide significance (*P* < 5 × 10^−8^) were extracted.^[22]^ Linkage disequilibrium (LD) was mainly determined by R^2^ = 0.001 and distance (kb = 10000).^[23]^ Using the R software Two-Sample MR package (version 4.2.2, R Foundation for Statistical Computing, Vienna, Austria) for the LD clump step, we finally extracted 44 SNPs from the IEU database and 14 SNPs from the FinnGen consortium database. An F-value is typically used for weak IV bias. When the F-value is <10, the selected genetic variant is a weak instrumental variable, which will bias the results.^[24]^ These values are calculated by: F=(N−k−1)/k∗R2/(1−R2), R2   =2∗(1−MAF)∗MAF∗ β 2/SD2, (N: sample size; k: number of IV; β: effect size; minor allele frequency; standard deviation = SE*√N; standard error). In this study, the F-values of all SNPs were calculated, and all F-values >29.9 were considered highly correlated with exposure and would not cause bias^[25,26]^ (Details of SNPs are provided in Supplementary Table 3, http://links.lww.com/MD/J513).

### 2.4. Statistical analysis

Two-sample MR was used to evaluate the causal relationship between exposure and outcome through the association of SNP exposure and outcome.^[27]^ The Wald ratio method was used first to evaluate the association between IBS and mental and psychological factors, such as stress, anxiety, and depression. This potential causal association was assessed using inverse variance weighting (IVW). For IVW, the existence of the intercept term was not considered in regression, and the reciprocal of outcome variance was used as the weight for fitting.^[28]^ Therefore, we calculated the weighted average values of the causal effect value of all IVs and used R software to convert them into odds ratios [OR].^[29]^ We also conducted a reverse MR analysis of the potential effects of IBS on stress, anxiety, depression, and other mental and psychological factors. Only if the premise that SNPs are all valid IV (absence of pleiotropy between genetic variants) was met couldn’t the causal estimation of IVW be biased. Therefore, this study also used MR-Egger regression, weighted median, penalty-weighted median estimation, simple mode, MR-PRESSO, and weighted mode for supplementary analyses.

The difference between the MR-Egger regression^[30]^ and IVW is that in the case of pleiotropy of IV (considering the existence of the intercept term), the linear function of the MR-Egger regression is weighted and fitted. The MR-Egger regression intercept term is used to evaluate the magnitude of horizontal pleiotropy indirectly, and the slope of MR-Egger regression is used to estimate the causal effect value; however, MR-Egger regression should meet the instrument strength independent of the direct effect (InSIDE), assuming that IVs are independently associated with exposure and outcome.^[31]^ The weighted median method can provide stable estimates even if up to 50% of genetic variation is invalid IVs.^[32]^ However, the potential bias caused by limited samples needed correction with the penalty-weighted median estimation.^[33]^ Heterogeneity was assessed by calculating the Cochran Q value, and horizontal pleiotropy was indirectly assessed by the MR-Egger intercept and MR-PRESSO. The sensitivity analysis of each SNP was performed using the leave-one-out method. A 2-sided *P* value of <.05 was considered statistically significant, and all the above statistical analyses were performed using the Two-Sample MR Data and MR-PRESSO package in R software, version 4.2.1.

This study followed the Strengthening the Reporting of Observational Studies in Epidemiology Using MR (STROBE-MR) Report Guide (Supplementary Table 4, http://links.lww.com/MD/J514).

## 3. Results

### 3.1. Instrumental variable

In this study, 58 independent SNPs (F-value ranged from 29.94 to 66.10) were obtained according to the above criteria and after removing LD, which was not prone to weak instrument bias and could better assess causality. The harmonious function was then used to align the effect allele of IV, and 4 SNPs with a palindromic structure were deleted: rs34555420, rs10143492, rs2876520, and rs393488. Therefore, 54 SNPs were included in the analysis.

### 3.2. Causal association by MR analysis

**Figure 2.**
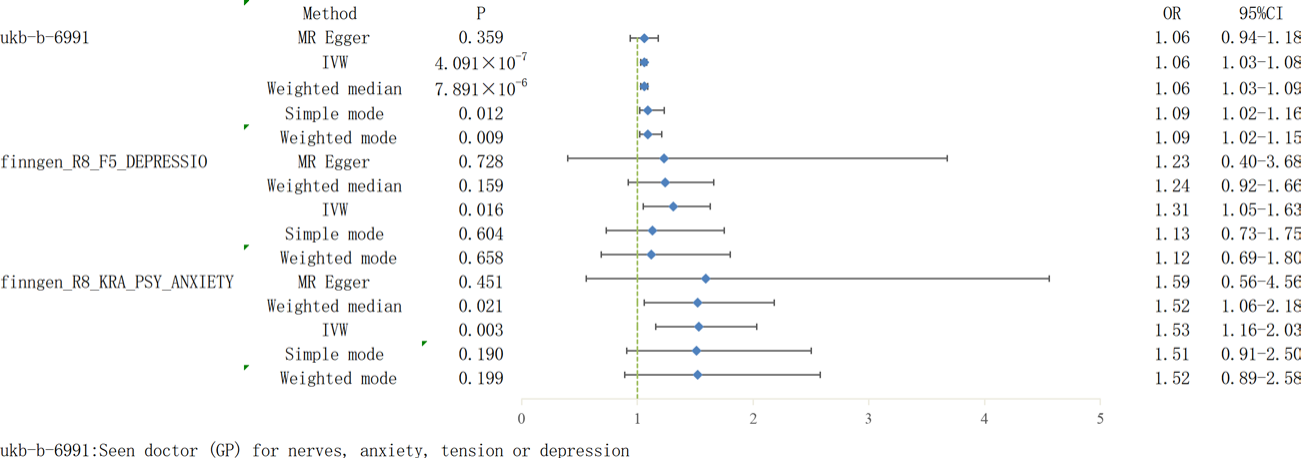
Forest plot: Results of Mendelian randomization of 5 causal analysis methods.

**Figure 3.**
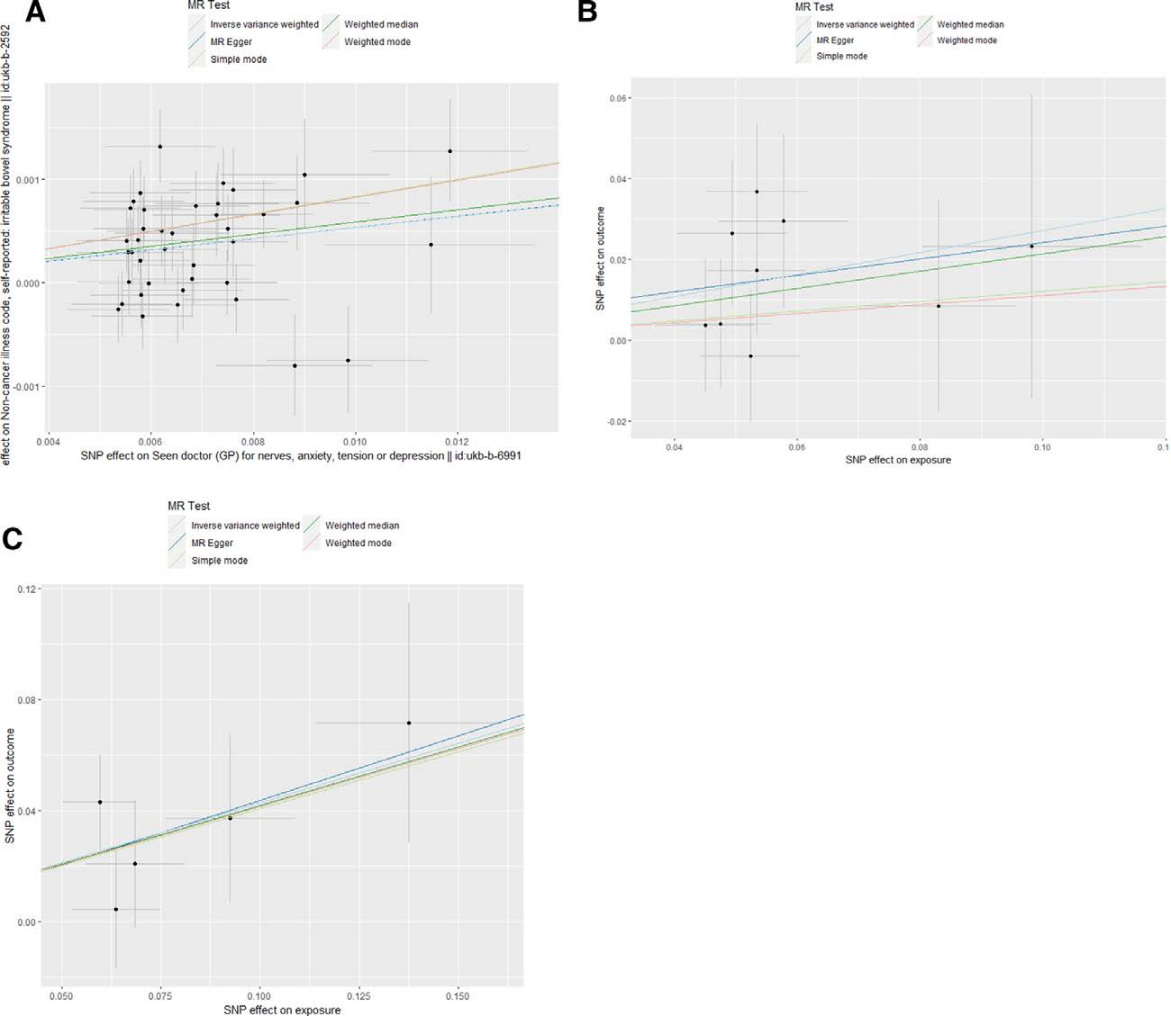
Scatter plot depicting tension, anxiety, depression and other adverse mood and mental illness and irritable bowel syndrome. Each of these points represents an IV. The vertical and horizontal lines at the center of the dot represent 95%CI. The slope of the colored line represents the size of the causal relationship. (A) Seen doctor (GP) for nerves, anxiety, tension or depression, (B) anxiety disorders syndrome, (C) Depression. CI = confidence interval, IV = instrumental variables.

IVW was used as the main analysis method, and the IVW results were converted into OR values [stress, anxiety and depression, (OR = 1.06, 95% confidence interval [CI]: 1.03–1.08, *P* = 4.091 × 10^−7^], (Depression, [OR = 1.31, 95% CI: 1.05–1.63, *P* = .016]), (Anxiety, [OR = 1.53, 95% CI: 1.16–2.03, *P* = .003]) (as shown in Fig. 2), indicating that psychological factors such as stress, anxiety, and depression are causal and positively correlated with IBS (b > 0), and mental and psychological factors are potentially important risk factors for IBS (as shown in Fig. 3 and Supplementary Fig. 1, http://links.lww.com/MD/J515). Contrastingly, the reverse MR analysis showed no evidence that IBS had a causal relationship with stress, anxiety, depression, or other mental and psychological factors. The MR-Egger regression result was *P* > .05, which is not statistically significant. The additional median method, simple mode, and weighted mode were consistent with the IVW results (see Fig. 3 for details).

### 3.3. Sensitivity analysis of tension, anxiety, depression, and other mental and psychological factors and IBS

**Figure 4.**
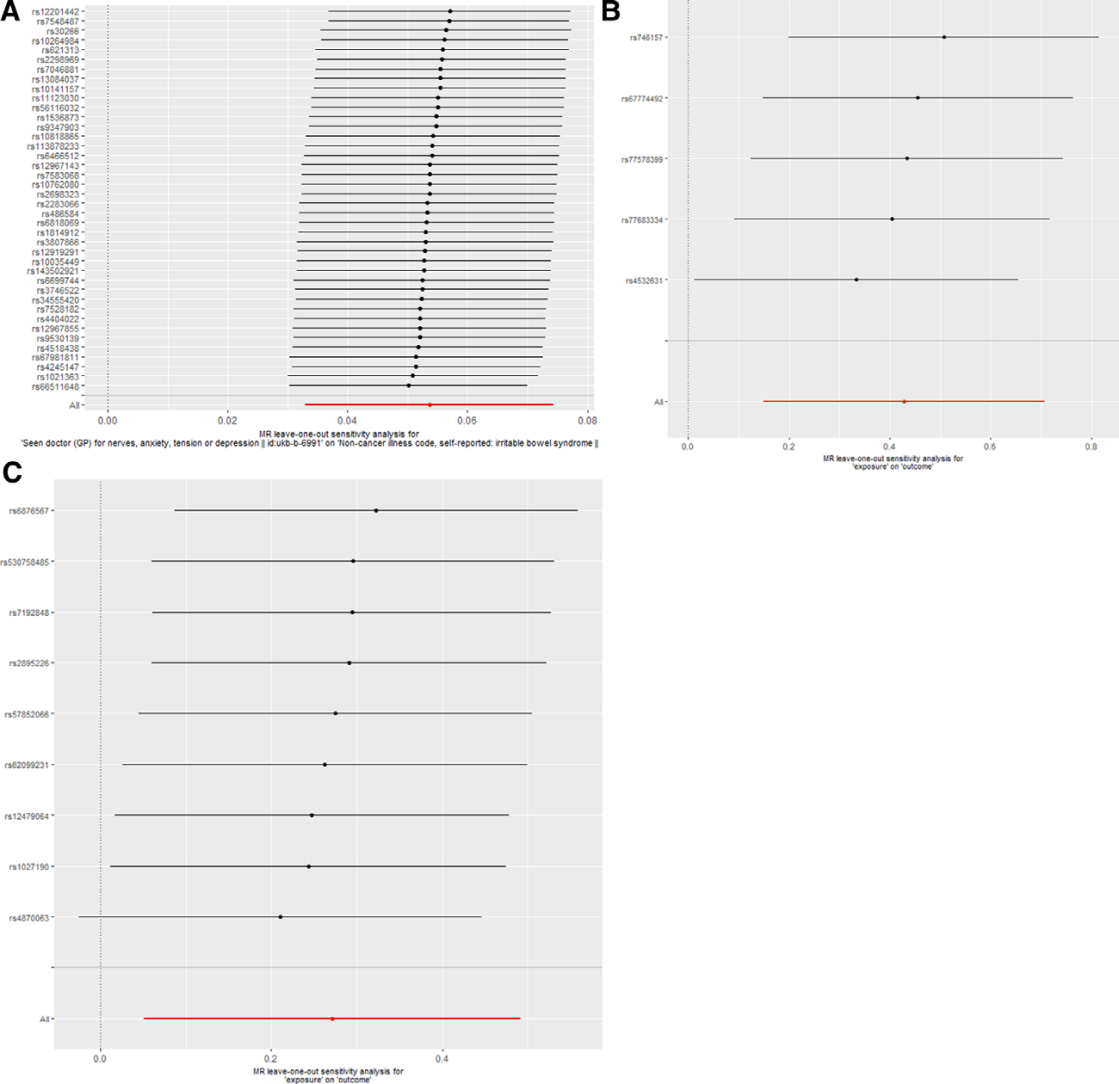
MR leave-one-out sensitivity analysis. MR: Mendelian randomization. The bars indicate the CI. Circles indicate that if each SNP is missing, MR assessment is performed using the fixed effects model IVW. (A) Seen doctor (GP) for nerves, anxiety, tension or depression, (B) anxiety disorders syndrome, (C) Depression. CI = confidence interval, IVW = inverse variance weighting, MR = mendelian randomization, SNP = single nucleotide polymorphism.

Heterogeneity was tested using the Cochran Q test. IVW analysis and MR-Egger regression showed that the *P* of the first group was much <.05 (Table 1), which indicates that there is potential heterogeneity among IVs. Therefore, we used a random effect model IVW analysis to estimate the causal effect value of MR. The data showed that there was a causal relationship between IBS and psychological factors such as stress, anxiety, and depression (*P* < .05). In addition, with an increase in the severity of mental and psychological factors, such as stress, anxiety, and depression, the risk of IBS also increased (b > 0). The remaining *P* values were all >.05, indicating that potential heterogeneity was unlikely. The results of the MR-Egger regression showed no evidence that the Egger-Intercept was statistically different from 0 (Table 1); therefore, there was no effect of potential pleiotropy between the involved SNPS and IBS (*P* = .993). Additionally, no obvious heterogeneity or horizontal pleiotropy was observed in the combined MR-presso results. When each SNP was used as an IV alone, the funnel plot (Supplementary Fig. 2, http://links.lww.com/MD/J516) showed that the results were symmetrically distributed, and no obvious outliers were found, indicating that bias was unlikely. We also performed leave-one-out sensitivity for each SNP analysis, which showed that the causal association between exposure and outcome did not change due to the influence of a single SNP, and all lines were located to the right of 0 (Fig. 4). Thus, we believed that the results of MR analysis were reliable.

**Table 1:** Description of the results of heterogeneity and pleiotropy analysis.

Exposures	Number of SNPs	MR-Egger		Cochran Q					MR-presso
		Egger_intercept	P-pleio	Method	Q	Q_df	Q-pval	P_mr	P
Seen doctor (GP) for nerves, anxiety, tension or depression	40	−3.407e-6	0.993	MR Egger	63.71	38	0.006	4.092E-07	0.018
				IVW	63.71	39	0.008		
Depression	9	0.004	0.905	MR Egger	4.784	7	0.686		0.778
				IVW	4.799	8	0.779		
Anxiety disorders syndrome	5	−0.003	0.949	MR Egger	2.407	3	0.492		0.695
				IVW	2.412	4	0.66		

IVW = inverse variance weighting, MR = mendelian randomization, SNP = single nucleotide polymorphism.

## 4. Discussion

The pathogenesis of IBS is still unclear, and its prevention and treatment are urgent problems to be solved.^[34]^ The MR method was first used to explore the causal relationship between IBS and psychological factors such as stress, anxiety, and depression. This study found that there may be a positive relationship between these factors and that the latter may increase the risk of IBS.

Previous studies have found that the pathogenesis of IBS is related to the gut-brain axis disorder, visceral hypersensitivity, psychological factors, inflammation, gastrointestinal motility, intestinal microbiota, intestinal mucosal permeability, and other factors,^[35]^ among which the gut-brain axis disorder plays an important role and has attracted increasing attention.^[36,37]^ The abdomen, known as the second brain, supports the gut-brain axis theory. The gut-brain axis is a 2-way pathway that integrates the brain and gastrointestinal nervous system.^[38,39]^ Adverse emotions, such as stress, anxiety, depression, and mental pressure, stimulate the gut-brain axis, which affects the autonomic nerve through the central nervous system. The increased secretion of corticotropin-releasing factor by the hypothalamus activates the hypothalamic-pituitary-adrenal (HPA) axis and transmits stimulation to the gastrointestinal nervous system.^[40]^ Thus, the stress in the gastrointestinal tract leads to the dysbiosis of the gastrointestinal flora, such as the reduction of diversity and the increase of intestinal epithelial permeability, which causes the immune response and visceral hypersensitivity changes, leading to the symptoms of IBS.^[41–43]^ Mounting evidence shows abnormal brain activation in the cerebral cortex of IBS patients with anxiety and depression, which is highly correlated with visceral pain and can lead to abdominal pain and other symptoms.^[44]^ Abnormal activation of brain areas, such as the prefrontal cortex and amygdala, can be observed under brain magnetic resonance imaging in patients with abdominal pain, abdominal discomfort, and other symptoms accompanied by depression and anxiety, as well as high intestinal sensitivity and reduced pain threshold during rectal distension test detected by cranial magnetic resonance imaging.^[45–48]^ An observational study found that gray matter density in the brain regions of patients with IBS was reduced due to anxiety and depression compared with that of healthy people; however, there was no difference in gray matter density between the experimental group and the control group after removing anxiety and depression.^[49]^ Stress factors, such as corticotropin-releasing factor and Adrenocorticotropic Hormone, increase when stress, depression, and anxiety occur, causing stress response and affecting gastrointestinal tract activity.^[50]^ In addition, pain can aggravate depression and anxiety through the pain matrix in the brain region.^[51]^ The gut-brain axis theory^[52–54]^ study found that gut microbiota is closely related to emotional factors such as stress, anxiety, and depression. At the same time, with the emergence of the bio-psycho-social medical model, stress, anxiety, depression, and other mental and psychological factors have gradually attracted the attention of scholars as independent pathogenic factors.^[55]^ A prospective study (n = 620) that followed up with participants after 10 months found that stress, anxiety, and depression were high-risk factors for IBS occurrence and led to a higher incidence of IBS.^[56]^ Sibelli et al conducted a meta-analysis of 11 studies on the relationship between stress, anxiety, and the incidence of IBS, finding that the probability of IBS in patients with anxiety was 2.38 times higher than that in healthy people (RR = 2.38, 95% CI: 1.58–3.60) and that in patients with depression was 2.06 times higher than that in healthy people (RR = 2.06, 95% CI: 1.44–2.96), which indicated that anxiety, depression or other negative emotions, and mental disorders were important risk factors for inducing IBS.^[57]^ Midenfjord et al also obtained similar results from a single-factor analysis of 769 patients.^[58]^ Therefore, mental and psychological factors such as stress, anxiety, and depression can lead to abnormal interaction of the HPA axis and further affect the nervous system of the brain and intestinal tract, causing the HPA axis to interfere with the normal physiological function of the intestinal tract and induce IBS.^[59]^

In addition, mental and psychological factors such as stress, anxiety, and depression may affect intestinal microecology and immune function to induce the onset and aggravation of IBS.^[60,61]^ Inflammatory factors such as Interleukin-1 (IL-1), C-reactive protein, and IL-6 are significantly increased in the peripheral blood of patients with anxiety and depression,^[62,63]^ suggesting that anxiety and depression may induce inflammation. One study showed that compared with patients without depression and anxiety, IBS patients with anxiety and depression have increased IL-1β in the intestinal mucosa, a pro-inflammatory factor that causes diarrhea,^[64]^ and significantly decreased IL-10, which has anti-inflammatory effects,^[65]^ resulting in an imbalance of the 2, thereby increasing the level of inflammation in the intestinal tract and inducing the onset and aggravation of IBS to a certain extent.^[66]^ Mental and psychological factors, such as stress, anxiety, and depression, can cause abnormal secretion of gastrointestinal hormones like vasoactive intestinal peptide and somatostatin in the human body,^[67]^ resulting in gastrointestinal motility disorders. Moreover, studies have shown that the symptoms of IBS are related to abnormal serotonin transporter (SERT) gene expression.^[68]^ Anxiety and depression may lead to high expression of 5-hydroxytryptamine (5-HT) through the abnormal expression of the SERT gene,^[69]^ and 5-HT, as an indispensable neurotransmitter of the gut-brain axis, plays a regulatory role in intestinal motility, sensation, and secretion;^[70]^ its abnormal expression and receptor binding to stimulate cholinergic neurons, increase the release of acetylcholine, leading to the stimulation of intestinal smooth muscle and the acceleration of intestinal peristalsis,^[71]^ further causing visceral hypersensitivity, pain threshold reduction, gastrointestinal motility changes and thus inducing the aggravation of IBS. Depression or anxiety may also affect intestinal microbial diversity. Experiments have shown that the beneficial effects of Bifidobacterium, Lactobacillus, and Faecalibacterium were greatly reduced^[72]^ but pathogenic *Escherichia coli* and *Clostridium difficile* increased,^[73,74]^ causing a microbial imbalance, which disrupts intestinal homeostasis, disrupts the intestinal barrier, and weakens intestinal flora colonization resistance. This is the key mechanism that induces IBS.

An increasing number of studies have demonstrated that^[75,76]^ antidepressant use can relieve the symptoms of IBS. The improvement of depression can increase the tolerance of rectal dilatation [r = -0.37, *P* = .008].^[77]^ Another meta-analysis showed that the effective rate of tricyclic antidepressants in treating IBS was 58%.^[78]^ Small doses of amitriptyline could relieve abdominal pain and diarrhea and effectively improve the quality of sleep and life among patients with IBS,^[79,80]^ and the application of citalopram 20 mg·d-1 when used for 3 weeks can shorten the days of abdominal pain and alleviate the degree of abdominal distension in patients with IBS.^[81]^ At the same time, mind-body therapy has attracted more and more attention, including mindfulness therapy,^[82]^ hypnosis,^[83]^ cognitive behavioral therapy (CBT),^[84]^ yoga,^[85]^ and other therapies and has a good effect on improving the symptoms of IBS as well as the quality of life of patients. In recent years, traditional Chinese medicine therapy, primarily acupuncture, traditional Chinese medicine, and moxibustion have shown excellent efficacy.^[86]^

This study had the advantage of large sample size and effectively avoided the influence of confounding factors through a 2-sample MR analysis. Therefore, we can draw a causal relationship between IBS and stress, anxiety, depression, and other mental and psychological factors. Furthermore, we used different sensitivity analyses to verify the consistency of our results. Therefore, this causal estimate is reliable.

Our study had some limitations. This study used MR analysis but lacked certain empirical and observational studies, and the conclusions drawn may not be consistent with other studies. Therefore, it is impossible to test this pathogenesis further. To avoid spurious associations that may have been caused by different populations, the populations included in our study were all European; however, the results may not necessarily apply to the entire population. The strong heterogeneity of this study may make the overall synthesis of the available data challenging.

## 5. Conclusion

Our research shows that stress, anxiety, depression, other bad moods, and psychological illnesses are potentially important pathogenic factors in IBS. This study provides relevant genetic evidence for the onset of IBS and provides reference suggestions for its prevention and treatment.

## Acknowledgments

We thank the GWAS database for providing publicly available data, and we thank all the GWAS staff. The Publisher note: All claims expressed in this article are solely those of the authors and do not necessarily represent those of their affiliated organizations, or those of the publisher, the editors and the reviewers. Any product that may be evaluated in this article, or claim that may be made by its manufacturer, is not guaranteed or endorsed by the publisher.

## Author contributions

**Data curation:** Jingzhi Zhang.

**Formal analysis:** Yufei Hu.

**Funding acquisition:** Yuxia Ma.

**Investigation:** Yufei Hu.

**Methodology:** Zhihao Diao, Fengzhao Liu.

**Resources:** Wenchang Xu.

**Software:** Zhihao Diao, Danyang Guo.

**Supervision:** Ruiyu Zhang.

**Validation:** Danyang Guo.

**Visualization:** Zhihao Diao.

**Writing – original draft:** Zhihao Diao.

**Writing – review & editing:** Yuxia Ma.

## Supplementary Material










