# Supplementary material for: Causal association between psycho-psychological factors, such as stress, anxiety, depression, and irritable bowel syndrome: Mendelian randomization
Source: Medicine (Baltimore). 2023 Aug 25;102(34):e34802. doi: 10.1097/MD.0000000000034802 (PMC10470701; doi:10.1097/MD.0000000000034802)
Supplement: Supplementary file 1 [file medi-102-e34802-s001.pdf]

Table S1. The data for the description of the contributing study were obtained from the IEU OpenGWAS project.

Trait	Seen doctor (GP) for nerves, anxiety, tension or depression	Non-cancer illness code, self-reported: irritable bowel syndrome
Sample Size (nCases/nControls)	459560 (158565/300995)	462933 (10939/451994)
Number of SNPs	9851867	9851867
Population	European	European
Sex	Males and Females	Males and Females
Release Date	2020-05-09	2020-05-10
Access address	https://gwas.mrcieu.ac.uk/datasets/ukb-b-6991/	https://gwas.mrcieu.ac.uk/datasets/ukb-b-2592/

Table S2. The data for the description of the contributing study were obtained from the FinnGen consortium.

Trait	Depression	Anxiety disorders syndrome	Irritable bowel syndrome
Sample Size (nCases/nControls）	338111 (38225/299886)	290361 （35385/254976）	284799 （8116/276683）
Population	European	European	European
Sex	Males and Females	Males and Females	Males and Females
Release Date	2022.01	2022.01	2022.01
Access address	https://storage.googleapis.com/finngen-public-data-r8/summary_stats/finngen_R8_F5_DEPRESSIO.gz	https://storage.googleapis.com/finngen-public-data-r8/summary_stats/finngen_R8_KRA_PSY_ANXIETY_EXMORE.gz	https://storage.googleapis.com/finngen-public-data-r8/summary_stats/finngen_R8_K11_IBS.gz
