# Supplementary material for: Causal association between psycho-psychological factors, such as stress, anxiety, depression, and irritable bowel syndrome: Mendelian randomization
Source: Medicine (Baltimore). 2023 Aug 25;102(34):e34802. doi: 10.1097/MD.0000000000034802 (PMC10470701; doi:10.1097/MD.0000000000034802)
Supplement: Supplementary file 2 [file medi-102-e34802-s002.pdf]

Table S3. Basic SNP information relevant to this study.

SNP	Chr	Position	EA	OA	EAF	Seen doctor (GP) for nerves, anxiety, tension or depression			Irritable bowel syndrome			R^2	F
						beta	SE	pval	beta	SE	pval		
rs10035449	5	164486067	C	T	0.533252	0.00585413	0.000977481	2.10E-09	0.000521047	0.00031575	0.0990011	7.80426E-05	35.86791945
rs10141157	14	104018105	C	T	0.513815	0.00579658	0.00097716	3.00E-09	-0.000115423	0.000315538	0.709999	7.65662E-05	35.18928287
rs10143492	14	75212114	C	G	0.545353	0.00625622	0.000980196	1.70E-10	0.000505302	0.000316545	0.11	8.86375E-05	40.73767909
rs1021363	10	106610839	G	A	0.643587	-0.00740736	0.00102059	3.90E-13	-0.000968487	0.000329584	0.00329997	0.000114613	52.67716533
rs10264984	7	117624373	T	C	0.409418	0.00583058	0.000994135	4.50E-09	-0.000324083	0.000321075	0.31	7.48442E-05	34.39781837
rs10762080	10	68397445	G	A	0.584876	-0.00562619	0.000992175	1.40E-08	-0.000298297	0.000320464	0.35	6.99648E-05	32.15513514
rs10818865	9	126509599	G	A	0.209045	0.00682631	0.00120174	1.30E-08	0.000173187	0.000388115	0.66	7.02067E-05	32.26629423
rs11123030	2	124976163	C	T	0.510908	-0.00595904	0.000977052	1.10E-09	7.26E-06	0.00031564	0.98	8.09357E-05	37.19763727

rs113878233	1	191143630	T	C	0.061019	0.0114703	0.00204987	2.20E-08	0.000366583	0.000662259	0.58	6.81279E-05	31.31086203
rs12201442	6	24267818	G	A	0.106529	0.00985306	0.00158252	4.80E-10	-0.000745158	0.000511239	0.14	8.43461E-05	38.76519296
rs12919291	16	13800430	C	G	0.18922	0.0072763	0.00124931	5.70E-09	0.000655077	0.000403383	0.1	7.38085E-05	33.92179855
rs12967143	18	53099012	C	G	0.699435	-0.00761209	0.00107088	1.20E-12	-0.000396509	0.000345779	0.25	0.000109935	50.52709428
rs12967855	18	35138245	G	A	0.6688	-0.00687351	0.00104168	4.20E-11	-0.000746823	0.000336377	0.0259998	9.47338E-05	43.53981665
rs13084037	3	49214066	A	G	0.774396	-0.00651283	0.0011683	2.50E-08	0.00021178	0.000377381	0.57	6.76175E-05	31.07626678
rs143502921	20	59827231	C	G	0.179546	0.00885943	0.00142321	4.80E-10	0.000773274	0.000459591	0.09200005	8.4313E-05	38.74999929
rs1536873	9	36994681	A	G	0.468978	0.00555937	0.000984187	1.60E-08	1.30E-05	0.000317893	0.97	6.94262E-05	31.90759125
rs1814912	8	124164983	C	T	0.325616	0.0062108	0.00106149	4.90E-09	0.000502124	0.000342812	0.14	7.44884E-05	34.23429465
rs2283066	7	126172521	C	T	0.3755	-0.00573733	0.00100863	1.30E-08	-0.000417164	0.000325778	0.2	7.04017E-05	32.35939

rs2298969	4	3186244	G	A	0.481488	-0.00535903	0.000977753	4.20E-08	0.000253169	0.000315871	0.42	6.53647E-05	30.04084691
rs2698323	3	157974243	C	T	0.415706	0.00554663	0.00098974	2.10E-08	0.000297777	0.000319696	0.35	6.83352E-05	31.40611796
rs2876520	6	142996618	G	C	0.468008	0.0060133	0.000987067	1.10E-09	0.000193171	0.000318844	0.54	8.07443E-05	37.10968367
rs30266	5	103972357	A	G	0.32822	0.00765851	0.00104038	1.80E-13	-0.000156805	0.000336017	0.64	0.000117899	54.18794581
rs34555420	6	26090270	T	G	0.097832	-0.00900836	0.00164304	4.20E-08	0.000567455	0.000637161	0.37	6.54071E-05	30.06029955
rs34555420	6	26090270	T	G	0.097832	-0.00900836	0.00164304	4.20E-08	-0.00105501	0.000530803	0.0479999	6.54071E-05	30.06029955
rs3746522	20	44680412	T	C	0.215906	0.00730671	0.00121043	1.60E-09	0.00076363	0.00039086	0.051	7.92844E-05	36.43866803
rs3807866	7	12250378	A	G	0.410746	0.0074939	0.000990903	3.90E-14	0.000526526	0.000319976	0.1	0.000124439	57.19415005
rs393488	9	17044971	A	T	0.467466	-0.00564139	0.000981676	9.10E-09	-0.000461224	0.000317103	0.15	7.18559E-05	33.02432972
rs4245147	11	113318007	T	C	0.514502	0.00579555	0.000984572	3.90E-09	0.000869638	0.000318021	0.00619998	7.5391E-05	34.6491402

rs4404022	15	74010430	T	A	0.4165532	0.00559119	0.000992331	1.80E-08	0.000722408	0.000320422	0.02399999	6.90754E-05	31.74632763
rs4518438	5	88157552	C	T	0.5101117	-0.00566112	0.000975737	6.60E-09	-0.000787219	0.000315201	0.01299999	7.32428E-05	33.66179603
rs486584	11	66358126	T	C	0.5237877	0.00552277	0.000977235	1.60E-08	0.000406506	0.000315619	0.2	6.94934E-05	31.93846195
rs56116032	9	11453149	G	A	0.2194522	-0.00662701	0.00118115	2.00E-08	7.28E-05	0.000381472	0.85	6.84941E-05	31.47916857
rs621313	11	88913663	G	A	0.49123	0.00748553	0.000979968	2.20E-14	-7.96E-07	0.000316533	1	0.000126947	58.34712099
rs6466512	7	114842574	A	G	0.4172265	-0.00578344	0.000992323	5.60E-09	-0.00021286	0.000320518	0.51	7.39081E-05	33.96756879
rs66511648	3	117515519	C	T	0.2847735	0.00617337	0.00108633	1.30E-08	0.00131992	0.000350911	0.00017	7.02665E-05	32.29380198
rs6699744	1	72825144	T	A	0.6159666	0.00819286	0.00100769	4.30E-16	0.000661388	0.000325603	0.04200001	0.000143818	66.10210349
rs67981811	6	28354835	G	C	0.11416	-0.0118552	0.00153202	1.00E-14	-0.00127768	0.000494974	0.00980009	0.000130213	59.84839744
rs6818069	4	131153170	G	T	0.2555475	0.00641818	0.001125	1.20E-08	0.000477029	0.000363374	0.19	7.08183E-05	32.54744118

rs7046881	9	82515992	G	T	0.4232242	-0.00543475	0.000990133	4.00E-08	0.000204776	0.000319775	0.52	6.55543E-05	30.12799161
rs7528182	1	67067369	T	C	0.4331777	0.00586087	0.00098508	2.70E-09	0.000707198	0.000318272	0.02599998	7.70204E-05	35.39804551
rs7548487	1	177025098	G	A	0.1162166	0.00880409	0.00152217	7.20E-09	-0.000798618	0.000491621	0.1	7.28345E-05	33.47410513
rs7583068	2	212634108	A	T	0.3196011	0.00626574	0.00104958	2.40E-09	0.00032842	0.000339096	0.33	7.75421E-05	35.63787139
rs9347903	6	165114404	T	C	0.2263144	0.00679779	0.00116505	5.40E-09	3.86E-05	0.000376284	0.92	7.40751E-05	34.04430476
rs9530139	13	31847324	T	C	0.1944144	-0.00760475	0.00123719	7.90E-10	-0.000902004	0.000399528	0.02399999	8.2209E-05	37.78289149

SNP	Chr	Position	EA	OA	EAF	Anxiety disorders syndrome			Irritable bowel syndrome			R^2	F
						beta	SE	pval	beta	SE	pval		
rs4532631	8	64671099	T	C	0.673668	0.0594839	0.00924865	1.26E-10	0.0430065	0.017121	0.0120077	0.000142443	41.36558676
rs67774492	15	69838971	A	G	0.142949	-0.0684242	0.012504	4.45E-08	-0.0208399	0.0229201	0.363222	0.000103119	29.94460133

rs746157	16	49559482	T	C	0.170903	0.0636758	0.0113128	1.82E-08	0.00454704	0.0213333	0.831215	0.0001091	31.68149084
rs77578399	6	51840614	T	C	0.0777216	-0.0924703	0.0162977	1.39E-08	-0.0374799	0.0303216	0.216429	0.000110867	32.19483552
rs77683334	12	22943353	C	T	0.0365296	-0.13758	0.0235171	4.91E-09	-0.0716753	0.043118	0.0964517	0.000117857	34.22473444

SNP	Chr	Position	EA	OA	EAF	Depression			Irritable bowel syndrome			R^2	F
						beta	SE	pval	beta	SE	pval		
rs1027190	10	104843328	T	G	0.722435	-0.0494412	0.00888066	2.59E-08	-0.0264888	0.017951	0.140047	9.16618E-05	30.99452255
rs12479064	2	99429576	C	T	0.16896	0.0578229	0.0105413	4.13E-08	0.0294095	0.0214959	0.171267	8.89843E-05	30.08907402
rs2895226	5	144460418	T	C	0.393459	0.0451102	0.00815972	3.23E-08	0.00377637	0.0164346	0.818261	9.03858E-05	30.56303113
rs4870063	6	151923512	C	A	0.654196	-0.0534065	0.0083326	1.46E-10	-0.0367795	0.0168447	0.0290028	0.000121483	41.0794478
rs530758485	3	43656252	G	A	0.107085	0.0829902	0.0126283	4.97E-11	0.00851927	0.026226	0.745302	0.000127717	43.18781898
rs57852066	16	72995680	G	A	0.0492467	0.0982292	0.0178809	3.94E-08	0.0232098	0.0375558	0.53657	8.92492E-05	30.17865612
rs62099231	18	53198529	G	A	0.459637	0.0533976	0.0080	2.53E-11	0.0173386	0.0160987	0.281472	0.000131617	44.50672738

							0401						
rs6876567	5	120759427	A	G	0.399705	0.0523901	0.00811811	1.09E-10	-0.00388613	0.0163733	0.812389	0.000123162	41.64722052
rs7192848	16	72173056	A	G	0.468658	0.0475714	0.00796549	2.34E-09	0.00415693	0.0160249	0.795324	0.000105478	35.66681284

SNP: single nucleotide polymorphism; CHR, chromosome; EA(effect allele), effect allele; OA(other allele): non-effector allele; EAF, effector allele frequency; beta: allelic effect value; SE: standard error.

SNP information from reverse MR Analysis.

SNP	chr	position	EA	OA	EAF	beta	SE	pval	R2	F
rs111344672	2	105849053	C	T	0.0050382	0.459135	0.0999376	4.3438E-06	7.62794E-05	21.10667535
rs115354969	1	240511027	G	A	0.0144785	-0.357638	0.074834	1.76096E-06	8.25413E-05	22.83948235
rs116870154	20	20699674	G	C	0.0400805	0.191302	0.0388374	8.40504E-07	8.76836E-05	24.26250208
rs118188507	16	75817202	C	T	0.0147492	0.313427	0.0617145	3.80111E-07	9.32128E-05	25.79260711
rs142706235	6	139356161	A	T	0.0382587	-0.202133	0.0446378	5.94593E-06	7.41061E-05	20.50528171
rs143609717	19	5114284	G	A	0.00888773	-0.438334	0.0975935	7.07473E-06	7.29045E-05	20.17276323
rs149378059	8	13968314	C	A	0.0974584	0.119619	0.0263752	5.75228E-06	7.43351E-05	20.56864452
rs1989447	2	56407651	C	A	0.243237	0.0923298	0.0184535	5.63352E-07	9.047E-05	25.03359097
rs34638686	3	48682658	C	T	0.123117	0.112674	0.0237175	2.02736E-	8.15627E-	22.56869601

								06	05	
rs4345097	3	117427801	T	C	0.781764	0.0913197	0.0197095	3.59915E-06	7.75821E-05	21.46716028
rs569542	1	111296652	A	G	0.826554	-0.0976391	0.0207022	2.40104E-06	8.03891E-05	22.24392367
rs62541875	9	16841757	A	G	0.1203	0.109574	0.0240765	5.33691E-06	7.48537E-05	20.7121501
rs7016453	8	130234167	G	A	0.223643	0.0836269	0.018889	9.54267E-06	7.08371E-05	19.6006703
rs7080005	10	134777790	G	A	0.973799	0.266801	0.0545205	9.90102E-07	8.65436E-05	23.94705473
rs72831330	17	4339554	C	G	0.210283	-0.0890514	0.0200937	9.3448E-06	7.0982E-05	19.64077117
rs73029069	3	11983573	C	T	0.135736	-0.107636	0.0239505	6.98731E-06	7.29914E-05	20.19681056
rs73267275	16	85555728	T	A	0.00385299	0.51099	0.111353	4.45502E-06	7.61036E-05	21.05803383
rs74687761	18	21772720	G	C	0.0505293	0.166207	0.0351395	2.24595E-06	8.08518E-05	22.37197507
rs74795993	15	37172343	C	A	0.0527515	0.15886	0.03462	4.46077E-06	7.60956E-05	21.05580501
rs75181063	2	241359547	C	A	0.0380013	-0.211504	0.0448798	2.44495E-06	8.02635E-05	22.20916371
rs7547481	1	39450334	T	C	0.0577929	0.15689	0.033383	2.60567E-06	7.9822E-05	22.08699647
rs77122573	17	61300675	G	A	0.00230633	0.637685	0.142049	7.14974E-06	7.28319E-05	20.15268432
rs79296260	18	57213196	T	C	0.0342874	0.19036	0.0425029	7.50793E-06	7.24937E-05	20.05908536

rs80296482	13	69982952	C	T	0.0140216	-0.337585	0.0751698	7.09022E-06	7.28896E-05	20.16862791
rs8039669	15	102068454	G	A	0.0419208	-0.191713	0.0419738	4.93696E-06	7.53931E-05	20.86139804
rs892023	19	19663850	A	G	0.382554	0.0776462	0.0163483	2.0392E-06	8.15225E-05	22.55755765
rs9583337	13	109768653	G	A	0.331668	0.0809941	0.0168733	1.58551E-06	8.32699E-05	23.04111928
rs9590023	13	95147135	T	G	0.363492	-0.0768453	0.0167858	4.69451E-06	7.57417E-05	20.9578821
rs10512344	9	109009495	C	G	0.042239	0.00434027	0.00078825	3.69999E-08	6.54876E-05	30.31821713
