# Supplementary material for: Causal association between psycho-psychological factors, such as stress, anxiety, depression, and irritable bowel syndrome: Mendelian randomization
Source: Medicine (Baltimore). 2023 Aug 25;102(34):e34802. doi: 10.1097/MD.0000000000034802 (PMC10470701; doi:10.1097/MD.0000000000034802)
Supplement: Supplementary file 4 [file medi-102-e34802-s004.pdf]

**Figure S1.** Forest plot of Wald ratio analysis results for each SNP. Each horizontal line represents the result estimated for each SNP using the Wald ratio method. The solid line completely on the left indicates negative correlation, and the realization completely shows positive correlation on the right. The solid line through the 0 baseline indicates that the result is not significant.

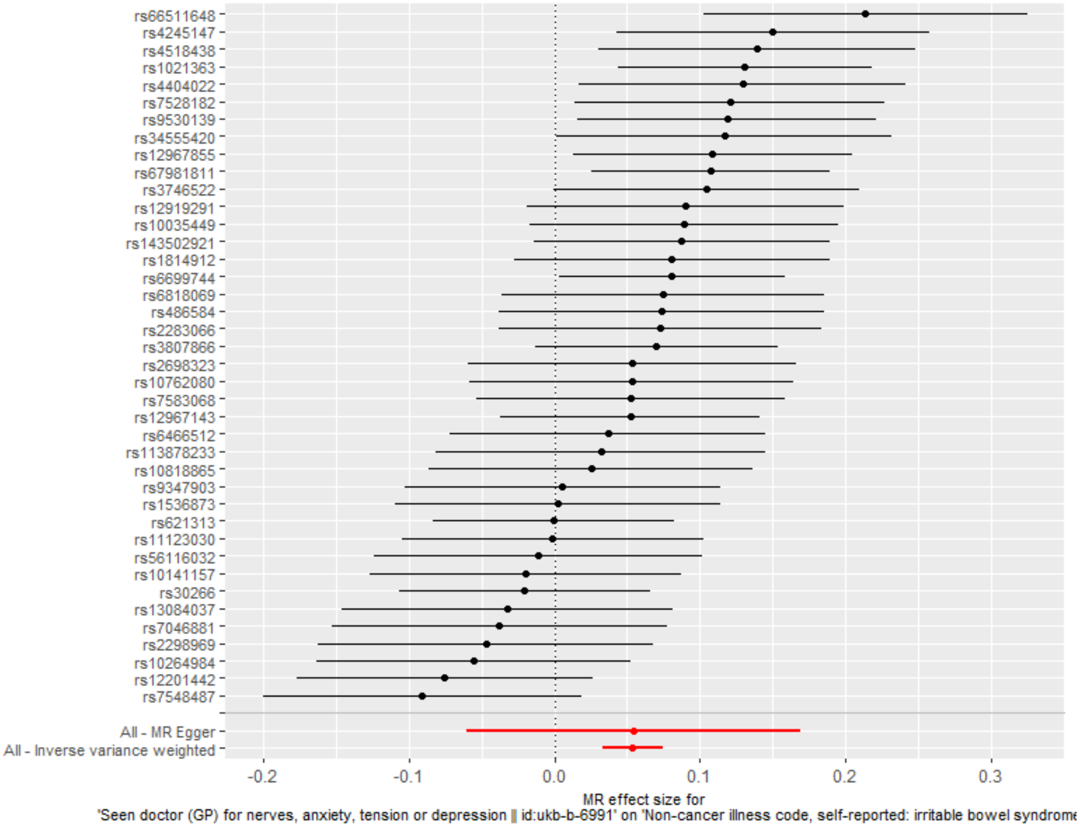


Seen doctor (GP) for nerves, anxiety, tension or depression

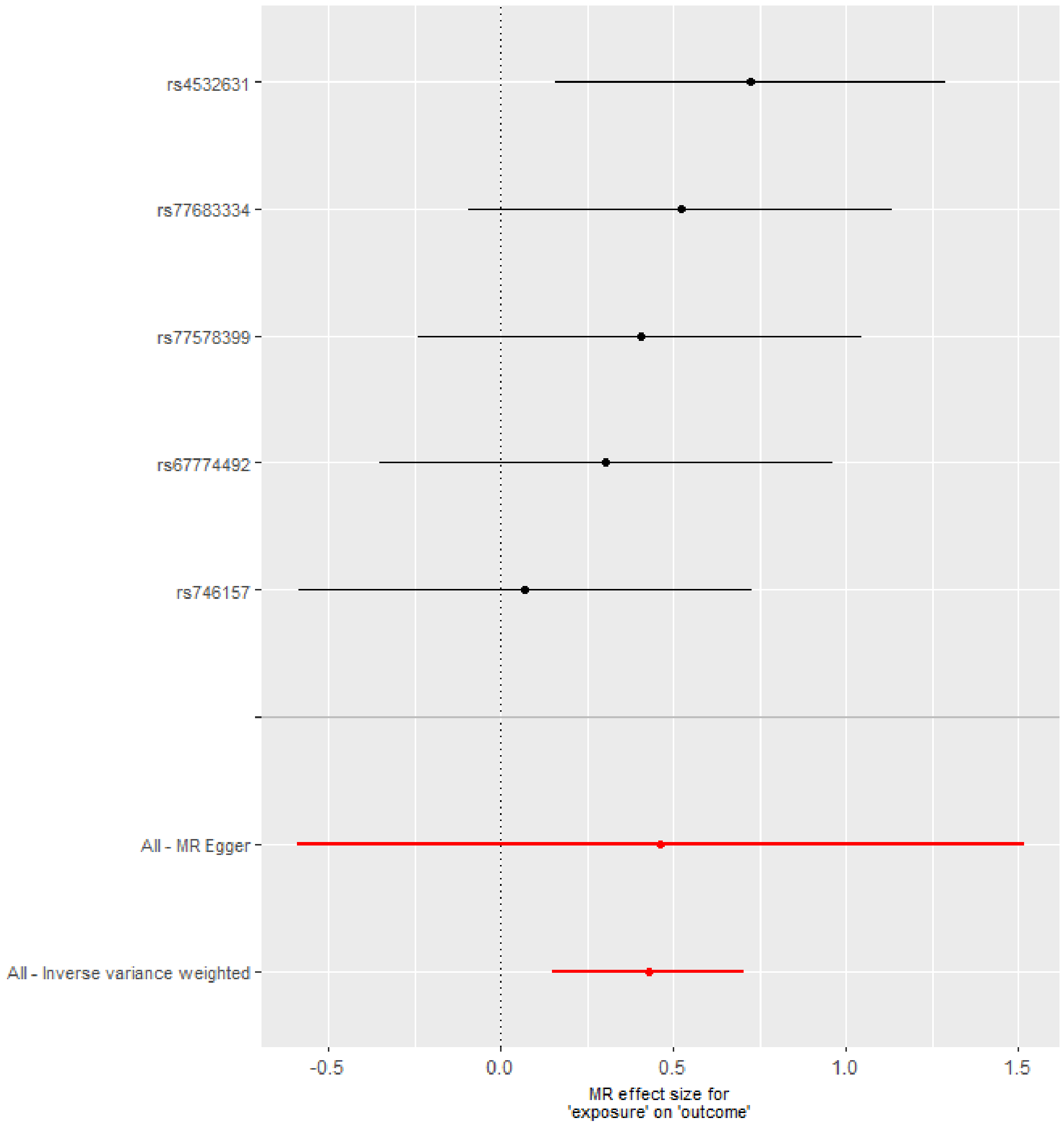


Anxiety disorders syndrome

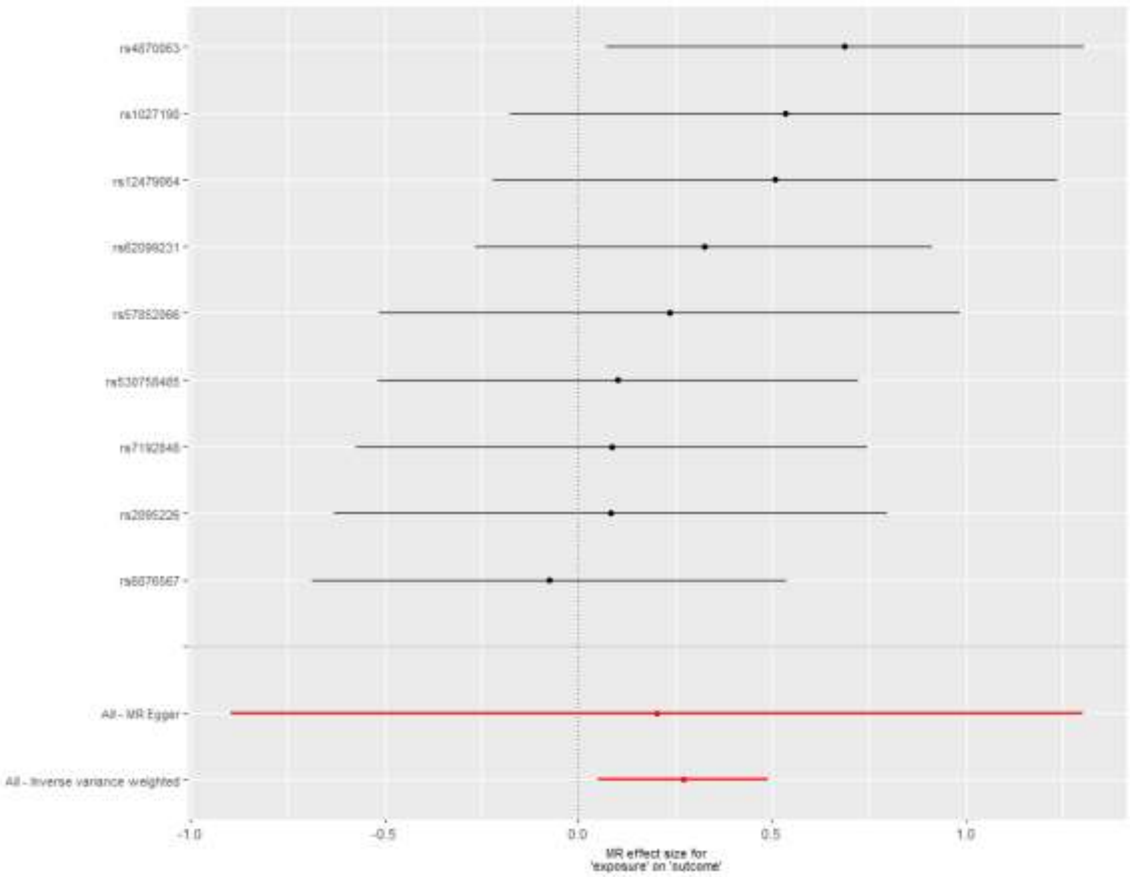
All - MR Egger
All - Inverse variance weighted
-1.0
-0.5
0.0
0.5
1.0
MR effect size for
'exposure' on 'outcome'

Depression
