# Supplementary material for: Causal association between psycho-psychological factors, such as stress, anxiety, depression, and irritable bowel syndrome: Mendelian randomization
Source: Medicine (Baltimore). 2023 Aug 25;102(34):e34802. doi: 10.1097/MD.0000000000034802 (PMC10470701; doi:10.1097/MD.0000000000034802)
Supplement: Supplementary file 5 [file medi-102-e34802-s005.pdf]

Figure S2. Mendelian randomization funnel plot between stress, anxiety, depression and other adverse mood and mental illness and IBS.

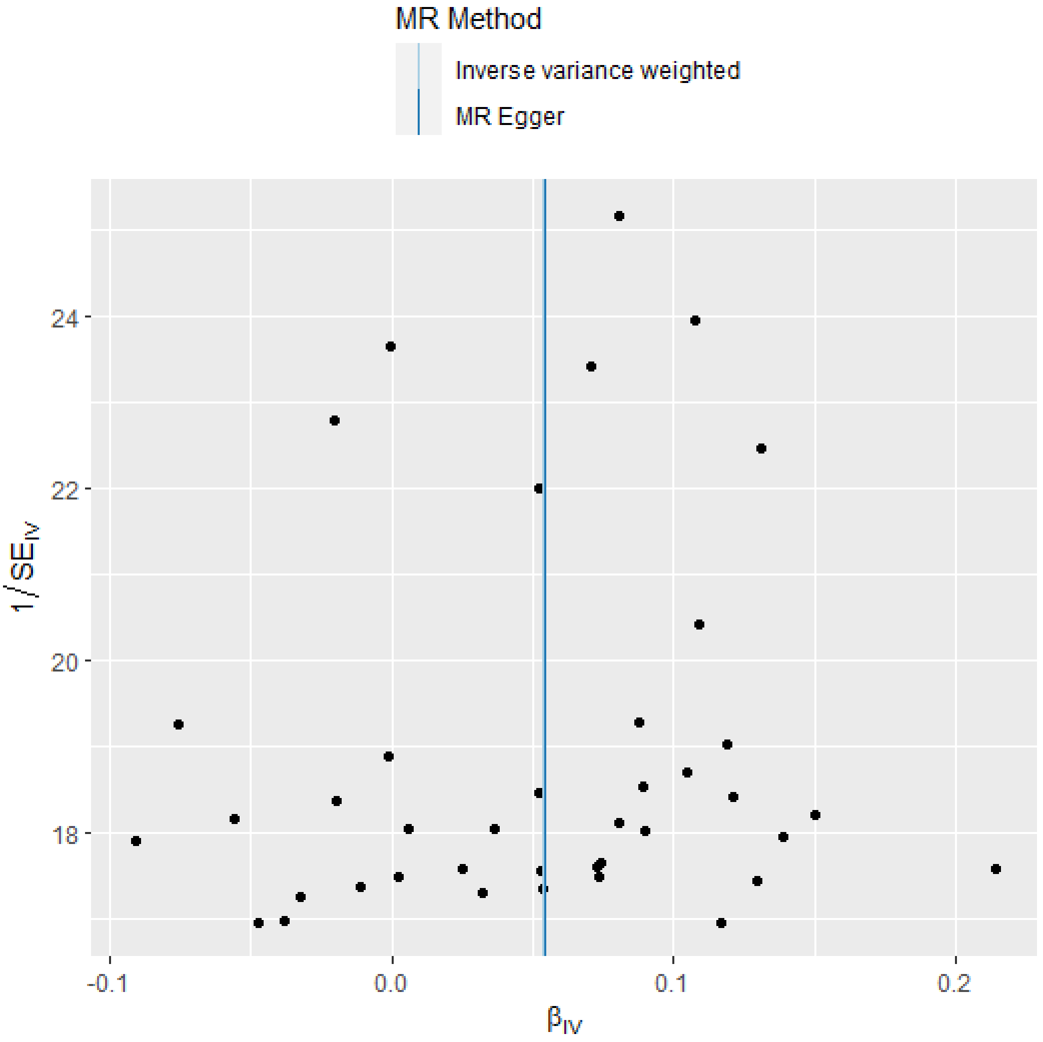

Seen doctor (GP) for nerves, anxiety, tension or depression

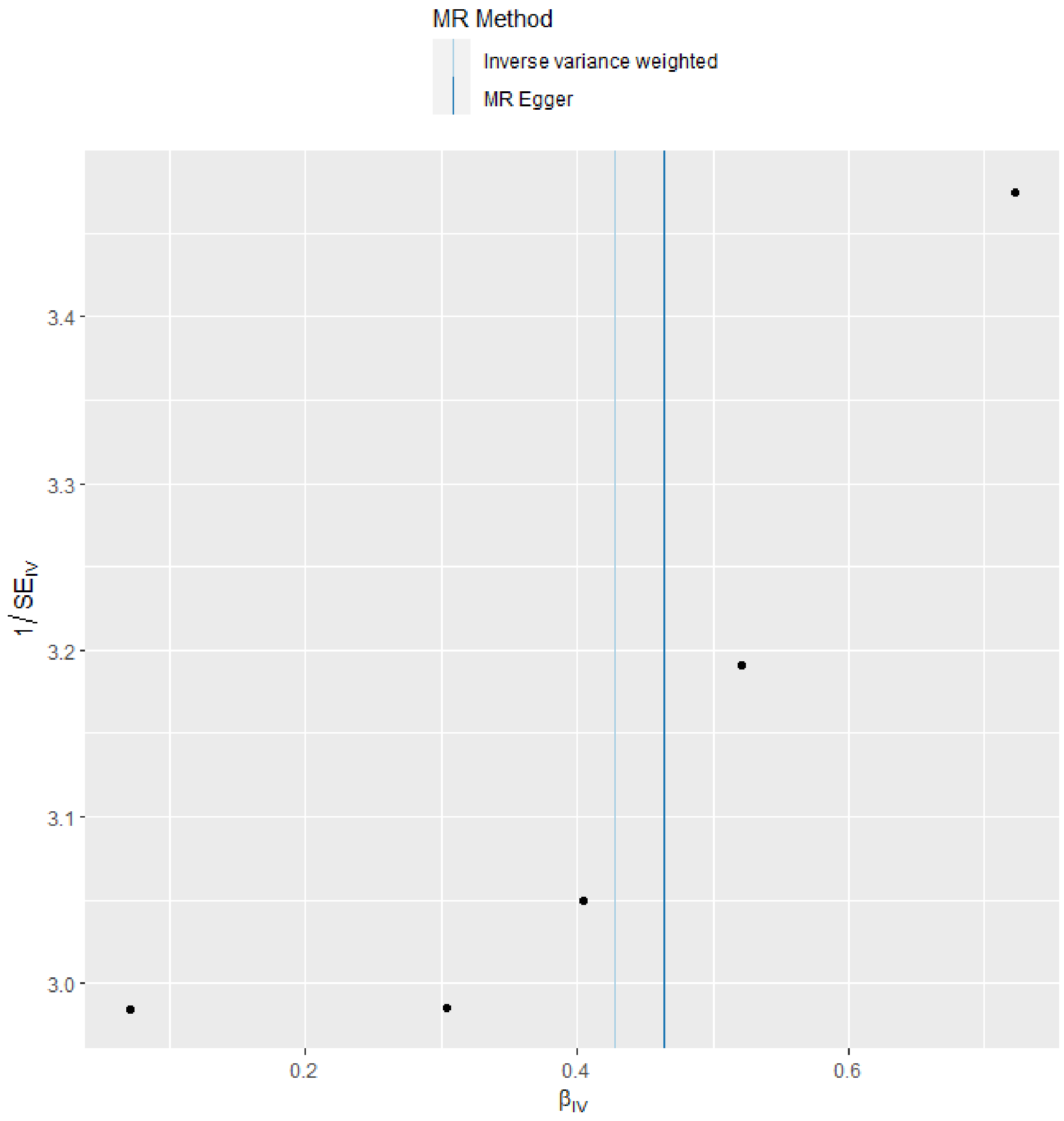

Anxiety disorders syndrome

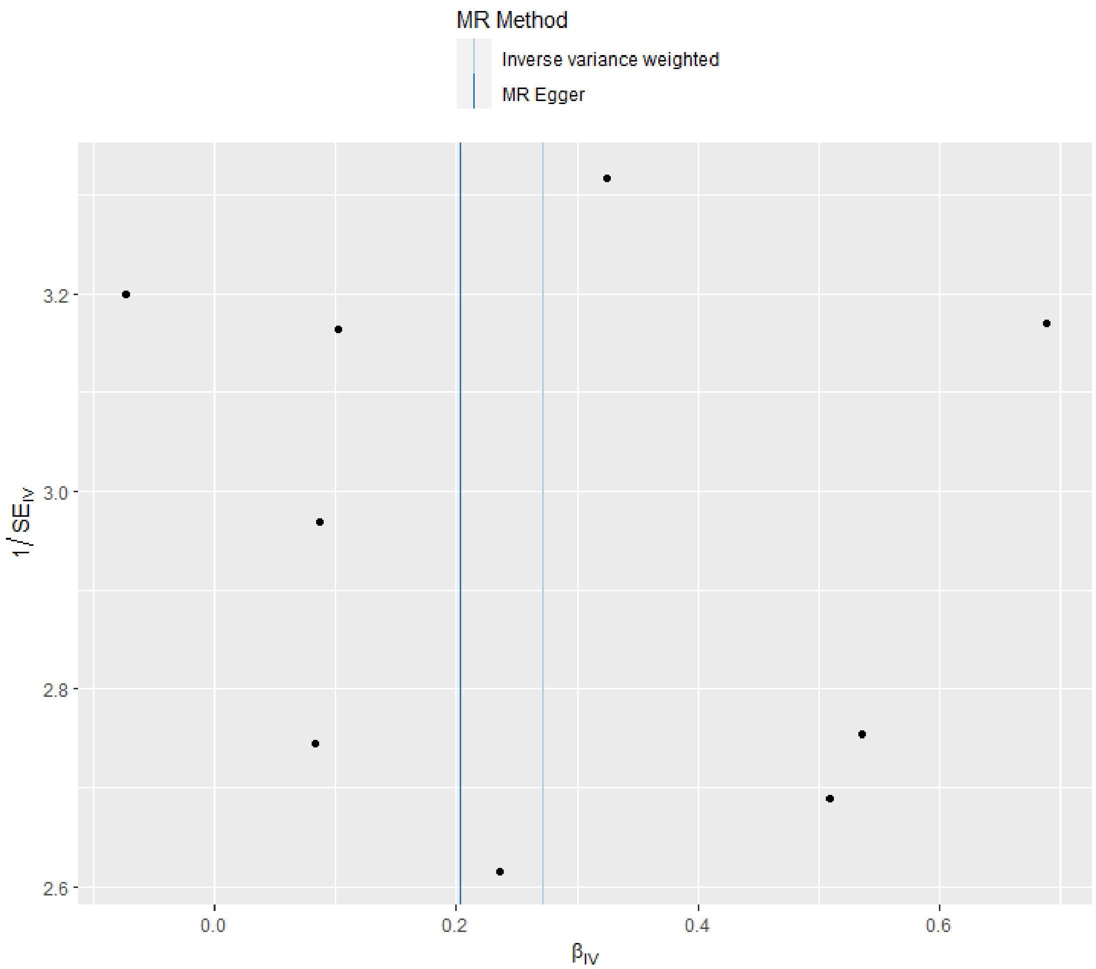
MR Method
Inverse variance weighted
MR Egger
3.2
3.0
2.8
2.6
0.0
0.2
0.4
0.6
β_{IV}
$1/SE_{IV}$

Depression
